# Imaging Cell Surface Plectin in PDAC Patients – A First-In-Human Phase 0 Study Report

**DOI:** 10.1007/s11307-025-02001-8

**Published:** 2025-03-26

**Authors:** Julien Dimastromatteo, Jiang He, Reid B. Adams, Kimberly A. Kelly

**Affiliations:** 1 Department of Biomedical Engineering, University of Virginia, Charlottesville, VA USA; 2 Department of Radiology and Medical Imaging, University of Virginia, Charlottesville, VA USA; 3 Department of Surgery, University of Virginia, Charlottesville, VA USA

**Keywords:** Cell Surface Plectin (CSP), Pancreatic cancer, Targeted therapy, Molecular imaging

## Abstract

**Purpose:**

Plectin is traditionally an intracellular cytoskeletal protein that maintains cell structure and stability. However, we and others have identified its surface-localized form in cancer (CSP), where it influences cell adhesion, migration, immune response, and tumor signaling. CSP-positive tumors (pancreatic, lung, ovarian, and breast cancers) contribute to over 3 million annual deaths, highlighting its clinical relevance. This phase 0 study aimed to evaluate PTP-01’s ability to target CSP in pancreatic tumors, despite their dense desmoplastic stroma, and to estimate CSP density and tumor vascularity.

**Methods:**

Pancreatic cancer patients (*n* = 3) received an intravenous injection of 100 µg PTP-01 labeled with 370 MBq ^111^In one day before resection. Whole-body planar scintigraphy and SPECT imaging were performed at multiple time points. Resected tumors and adjacent tissues were collected 28 h post-injection. Blood and urine samples were obtained for pharmacokinetic analysis. Tissue biodistribution was assessed using whole-body SPECT scans.

**Results:**

PTP-01 injection caused no reported adverse events. Uptake was primarily observed in the kidneys, liver, and bladder, with some tumor uptake. CSP density in tumors was estimated at 10⁶ molecules per cell. The elimination half-life (T₁/₂) ranged from 5 to 22 h across patients.

**Conclusion:**

PTP-01 imaging of pancreatic tumors revealed the ability of a targeted agent to bind to CSP. Further, CSP density in tumors was estimated to be on par with other surface molecules such as Her2 with effective targeted therapies. This study suggests that CSP is a highly expressed, accessible molecule for the development of targeted therapies such as antibodies or antibody–drug conjugates.

Pancreatic cancer is the third leading cause of cancer-related death in the United States, with a median survival of 6 months and a 5-year-survival rate of 12% [1]. Due to a lack of specific symptoms and early detection modalities, most patients are diagnosed with metastatic late-stage disease, for which 97% of patients will die within 5 years [1]. Therefore, to improve patient outcomes, it is critical to establish better imaging that shifts patient diagnosis earlier and new therapeutic strategies capable of targeting cancer cells at an early disease stage.

While genetic alterations have been widely explored for target discovery, cancer cells also exhibit an altered cell surface proteome, offering an underutilized avenue for early imaging and targeted therapy. In 2008, a phage display proteomic approach identified a peptide (KTLLPTP) that selectively bound to pancreatic cancer cells in an orthotopic murine model [2]. Proteomic analysis identified that KTLLPTP binds to plectin. In physiologically healthy cells, plectin is a 500 kDa scaffolding protein present in the cytoplasm and inner leaflet of the plasma membrane [3, 4]. Subsequent studies confirmed that plectin is an abundant cell surface target in malignant tissue that remains cytoplasmic in healthy tissue [5–9]. The surface-localized version of plectin (CSP) provides exceptional specificity, enabling targeted agents to bind tumor cells while bypassing intracellular plectin in healthy cells. A monoclonal antibody against CSP is currently in therapeutic development [10]. CSP has subsequently been found to be present on the surface in other tumors and has been recently reviewed as a biomarker and therapeutic target in cancer [3, 11–13].

The CSP-targeting peptide KTLLPTP has been adapted for numerous applications as a targeting ligand for imaging agents. In particular, preclinical studies using KTLLPTP labeled with ^99m^Tc and SPECT imaging of murine tumor models demonstrated a tumor accumulation and favorable biodistribution in addition to fast blood clearance [14]. Tumor models with higher CSP levels also had correspondingly high tumor uptake of ^99m^Tc-KTLLPTP. Here we report the results of a phase 0 trial examining a tetrameric version of KTLLPTP labeled with ^111^In called PTP-01 (NCT01962909) in patients with resectable pancreatic cancer.

The aim of this phase 0 clinical study was to assess the biodistribution and pharmacokinetics of PTP-01 as well as evaluate how well PTP-01 could reach pancreatic tumor cells in humans. The study was also designed to estimate the density of CSP per cell and vascularity of the tumor.

## Materials and Methods

### Patient Selection

Three patients (Pt 001, Pt 002, and Pt 003) with pancreatic cancer scheduled for surgical resection were enrolled in this exploratory phase 0 trial, designed to assess the feasibility, biodistribution, and pharmacokinetics of PTP-01 in a first-in-human setting. Inclusion criteria required normal kidney function, ECOG performance status 0–2, and informed consent**.** Exclusion criteria included impaired renal function, participation in another investigational drug study within 30 days, known allergies to study components, and serious medical conditions such as uncontrolled infections or cardiovascular disease. Pregnant or breastfeeding women were also excluded. This study was conducted over 10 years ago, during which comprehensive clinical data—including patient demographics, tumor staging, and preoperative imaging—were collected. However, due to the passage of time, much of this information is no longer accessible**.** All patients met clinical eligibility criteria for surgical resection, confirming the absence of distant metastases via standard preoperative imaging (CT and/or MRI). Diagnostic biopsies were performed as part of the standard clinical workup prior to surgery, and CSP expression was evaluated during the study. Currently, histological validation data are available for only one patient. The study was IRB-approved, and all patients provided written informed consent. Safety was monitored through clinical labs, electrocardiograms, vital signs, and physical exams up to seven days post-PTP-01 administration.

### PTP-01 Radiosynthesis and Quality Control

PTP-01 tetrameric peptide was synthesized using a multivalent scaffold-based conjugation approach, ensuring high binding affinity to CSP. The tetramer was generated by conjugating four CSP-targeting peptide units to a branched polyethylene glycol (PEG) linker, which enhances solubility and stability. This approach was chosen over the classical streptavidin–biotin method to improve pharmacokinetics and reduce potential immunogenicity. The synthesis was conducted in a GMP grade facility (CS Bio Company).

For radiolabeling, PTP-01 was labeled with ^111^In using 1,4,7,10-tetraazacyclododecane-1,4,7,10-tetraacetic acid (DOTA) as the chelator at Cardinal Health (Charlottesville, VA). DOTA was selected due to its well-characterized stability with ^111^In and its established use in clinical radiopharmaceuticals. Briefly, 444 MBq of ^111^In (Cardinal Health) was mixed with 120 µg of peptide at 40ºC for 60 min, then passed through a 0.2 µm syringe filter (PVDF) into a new sterile evacuated vial.

Radiochemical purity was determined using instant thin-layer chromatography (ITLC) with a mobile phase of 0.1 M sodium citrate buffer (pH 5.0) to ensure effective chelation. Additionally, a Sep-Pak membrane filtration system was used with gamma-well counting to measure the activity of the flow-through solution and Sep-Pak residue. Each dose was also tested for endotoxins to ensure safety. Radiochemical purity consistently exceeded 95%, confirming the stability and suitability of the radiolabeled agent for clinical use.

### PTP-01 Imaging Acquisition

#### *In Vivo* Planar Scintigraphy

Each patient received a single intravenous 100 µg bolus dose of PTP-01 labeled with 370 MBq ^111^In. Whole-body planar scintigraphy was performed at 10 min post-injection, followed by scans at 1, 2, 3, 4, 20, and 50 h. SPECT/CT imaging was performed at 4, 20, and 50 h post-injection using a Siemens Symbia™ T6 dual-head SPECT/CT system with low-energy high-resolution (LEHR) collimators optimized for ^111^In imaging. However, most of these data are no longer accessible due to storage limitations from the original study period. Quantitative analysis was conducted at the time using fused SPECT/CT images, and planar imaging remains available for biodistribution assessment. Tumor ROIs were initially defined using fused SPECT/CT images and cross-referenced with planar data for consistency. The CT component was acquired using a low-dose protocol without IV or oral contrast, primarily for anatomical localization and attenuation correction. However, attenuation correction was not applied to the SPECT images, as full quantitative SPECT reconstruction was not supported by the scanner at the time. Quantitative analysis (%ID) was derived from planar scintigraphy data using calibration factors from a standard source (syringe) imaged under identical conditions. While SPECT/CT facilitated ROI placement, quantitative measurements were based on planar scintigraphy due to the absence of attenuation-corrected SPECT data. After the final pre-surgical imaging session, surgery occurred 24 to 28 h post-injection**.** In some cases, post-surgical imaging—including planar scintigraphy and SPECT/CT (when available)—was performed at 50 h post-injection after patients recovered from anesthesia.

#### *Ex Vivo* Planar Scintigraphy

Following surgery, *ex vivo* planar images of the resected tumor and adjacent normal tissue (e.g., pancreas and duodenum) were obtained at 28 h post-injection. This timing aligns with surgical resection, allowing immediate imaging of fresh specimens. A white light image was collected for one patient to confirm tumor localization, but these images were not systematically obtained.

### ROI Selection and Quantification

Invicro® Imaging Services conducted the clinical trial image analysis, following the methods described by Siegel et al. [15] and Grimes et al. [16].

For *in vivo* images, we generated eight ROIs for each patient, including bladder, heart, left kidney, right kidney, liver, syringe, whole body, and tumor. Tumor ROIs were defined using fused SPECT/CT images to ensure precise anatomical localization, with adjustments made based on planar scintigraphy data for consistency across time points. Organ ROIs were manually created at the first time point and registered for all subsequent time points. Results were expressed as percent injected dose (%ID). Tumor-to-background ratios were calculated based on the concentration of PTP-01 in each region at each time point. In this study, quantitative measurements were not performed on normal pancreatic tissue. The primary focus was to assess tumor-specific uptake of PTP-01, and including normal pancreatic tissue was not part of the original ROI analysis protocol. We acknowledge this limitation, as tumor-to-normal tissue ratios could have provided additional context.

For *ex vivo* images, tumor ROIs were drawn using white light imaging, with the surrounding tissue considered background. Since the *ex vivo* data did not include a syringe, we applied syringe sensitivity values from the planar data analysis. The activity was calculated using planar data methods, ignoring the self-attenuation of the tissue because of tissue thickness.

### CSP Immunohistochemistry

Immunohistochemical staining for CSP was performed on a Discovery Ultra Staining Module (Roche Diagnostics, USA) by the Biorepository and Tissue Research Facility at the University of Virginia. Tissue Sects. (4 μm) were deparaffinized, and heat-induced antigen retrieval was conducted using Cell Conditioner 1 (Roche Diagnostics). Endogenous peroxidases were blocked before incubation with anti-plectin antibody (Abcam, ab32528). Detection was performed using OmniMap anti-rabbit multimer with DISCOVERY ChromoMap DAB Kit (Roche Diagnostics). Slides were counterstained with hematoxylin, dehydrated, cleared, mounted, and scanned using the Aperio ScanScope (Leica Biosystems, USA). However, due to the passage of time, only a limited number of stained tissue sections and corresponding analyses were available.

### CSP Density Estimation

The *in vivo* CSP antigen density was estimated using the Krogh cylinder model, which evaluates tissue microenvironmental factors such as vascularity from the quantitative imaging data [17, 18]. Capillary permeability (P), interstitial diffusivity (D), and void fraction were estimated based on molecular weight using empirical relationships derived from the literature [19].

The following input parameters were applied to all patients:

- Association rate constant (k_on_) = 1 × 10^5^ M^−1^ s^−1^
- Dissociation constant (K_D_) = 90 nM
- Molecular weight (MW) = 27.647 kDa
- Mass dose = 100 µg
- Internalization half-life = 13 h
- Residualization half-life = 120 h
- Bodyweight = 70 kg.

Blood clearance parameters were calculated individually for each patient based on a biexponential fit to blood %ID/g data. The parameters for patient Pt 001 were A = 0.492, t_1/2,α_ = 0.33 h, t_1/2,β_ = 6.4 h; for patient Pt 002, A = 0.338, t_1/2,α_ = 0.36 h, t_1/2,β_ = 11.5 h; for patient Pt 003, A = 0.423, t_1/2,α_ = 0.65 h, t_1/2,β_ = 9.6 h. CSP Antigen density was assumed to remain constant throughout the course of the simulation.

To determine CSP antigen density and vascularity multiple simulations were run, and the mean squared error (MSE) between the simulated and quantitative imaging data was calculated. The simulation with the lowest MSE was considered the best fit and the corresponding parameters were considered as the parameters of best fit.

### Pharmacokinetic Analysis

Blood and urine samples were collected at multiple time points post-injection to assess the pharmacokinetics of PTP-01. Blood samples were obtained at approximately 10 min, 1, 2, 4, 20, and 50 h post-injection to measure circulating activity and determine clearance rates. Additionally, urine samples were collected at corresponding time points to evaluate renal clearance and urinary excretion of PTP-01. The primary pharmacokinetic data were derived from direct blood and urine sample measurements.

Blood and urine data were converted to %ID/mL for blood and %ID for urine using counts per minute (CPM), decay-corrected injected dose converted to CPM, and sample weights (with 1 g = 1 mL). For urine, total sample weights were used, and a fixed hematocrit fraction (0.45 for men, 0.40 for women) and a gamma counter efficiency fraction of 0.278 were applied.

The total accumulated %ID of urine was computed under the assumption that all activity in the blood was located in the plasma. Blood pharmacokinetics were analyzed using two approaches:

1. **Non-compartmental analysis**: Data were fit to a single-exponential model, including at least three points based on goodness of fit, with the exclusion of C_max_.
2. **Biexponential model fitting**: Data were also fit to a biexponential distribution to evaluate early and late clearance phases.

All pharmacokinetic analyses were conducted using MATLAB software, which provided the best-fit curves and corresponding parameters.

## Results

### *In vivo *and *Ex vivo* Planar Scintigraphy

**Fig. 1:**
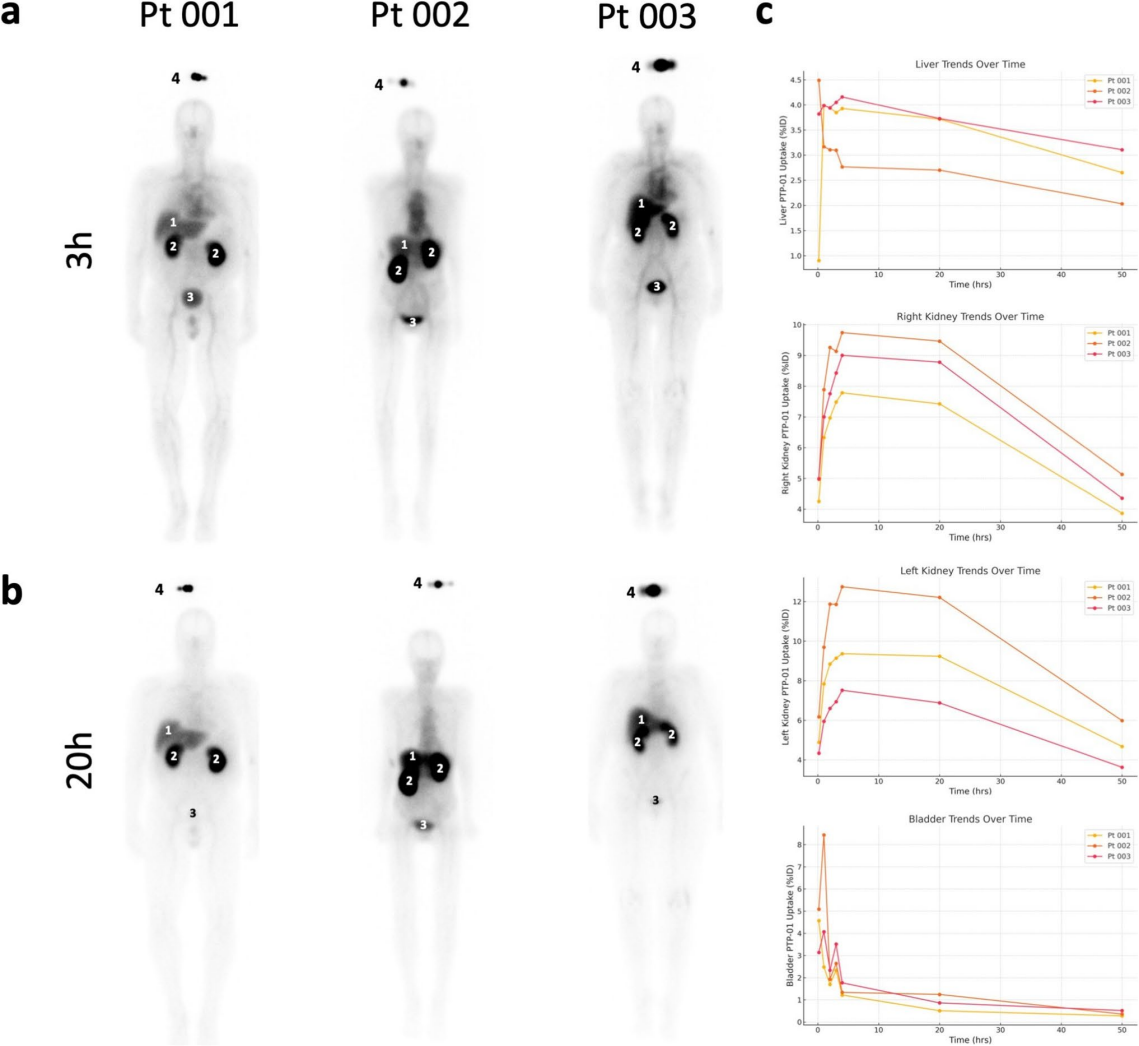
Planar imaging of PTP-01 at 3 and 20 h post-injection. **a** Whole-body planar scintigraphy images acquired 3 h after injection of ^111^In-labeled PTP-01. **b** Whole-body planar scintigraphy images acquired 20 h after injection. In both set of images, predominant uptake is seen in the kidneys, liver, and bladder. **c** Clearance organs PTP-01 uptake trends expressed in percent injected dose. 1 = liver; 2 = kidney; 3 = bladder; 4 = syringe

Whole-body planar scintigraphy following PTP-01 injection showed predominant uptake in the kidneys, liver, and bladder (Fig. 1a and b). At 3 h post-injection, intense uptake was observed in these clearance organs, which persisted at 20 h post-injection with reduced intensity due to partial clearance (Fig. 1a, b). PTP-01 uptake trends in these organs over time are depicted in Fig. 1c, expressed as %ID.

**Fig. 2:**
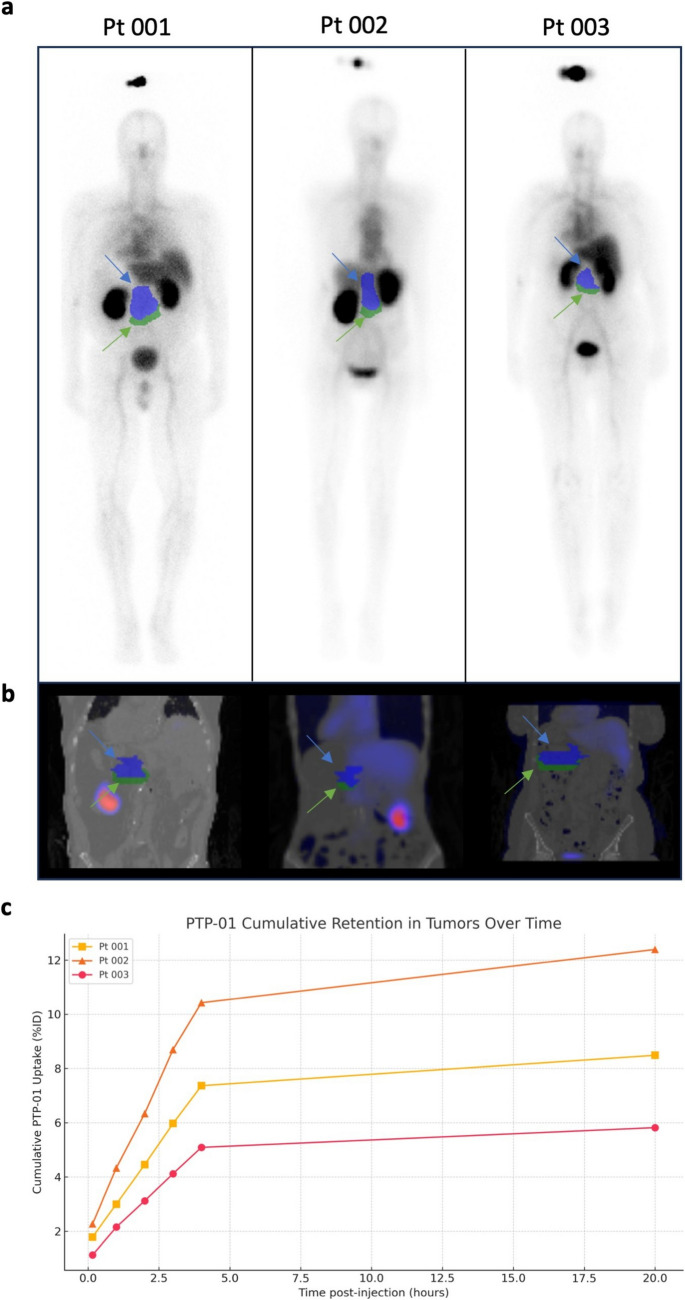
PTP-01 uptake in tumors. **a** Whole-body planar scintigraphy images of the three patients following PTP-01 injection. The syringe containing the radiotracer is visible above each patient’s head, serving as a reference for quantification. Blue arrows indicate the regions of interest (ROIs) corresponding to the tumor, while green arrows point toward adjacent normal tissue, which was not quantified. **b** Longitudinal sections of fused SPECT/CT images for each patient, showing the same ROIs as in panel a. Tumor delineation is highlighted with blue arrows, and adjacent normal tissue is marked with green arrows, confirming accurate anatomical localization and functional uptake of PTP-01. This approach ensured precise tumor identification based on both anatomical (CT) and functional (SPECT) data. **c** Cumulative retention of PTP-01 in tumors over time, expressed as % injected dose (%ID). The graph illustrates consistent PTP-01 accumulation in the tumor regions across all patients, supporting the specificity of PTP-01 for CSP-expressing pancreatic tumors

Due to high kidney uptake, tumor visualization on planar images was initially challenging. However, tumor regions of interest were accurately delineated using fused SPECT/CT images, with corresponding ROIs applied to planar scintigraphy for quantitative analysis (Fig. 2a, b). Whole-body planar images show the syringe above each patient's head as a reference for activity quantification, with blue arrows indicating tumor regions and green arrows marking adjacent normal tissue. Although SPECT/CT imaging was conducted at multiple time points (4, 20, and 50 h post-injection), these data are no longer accessible due to storage limitations from the original study period. However, quantitative analysis of the *in vivo* planar scintigraphy data revealed specific tumor uptake, with an average cumulative retention of 8.90 ± 3.31%ID across all patients (Fig. 2c).

**Fig. 3:**
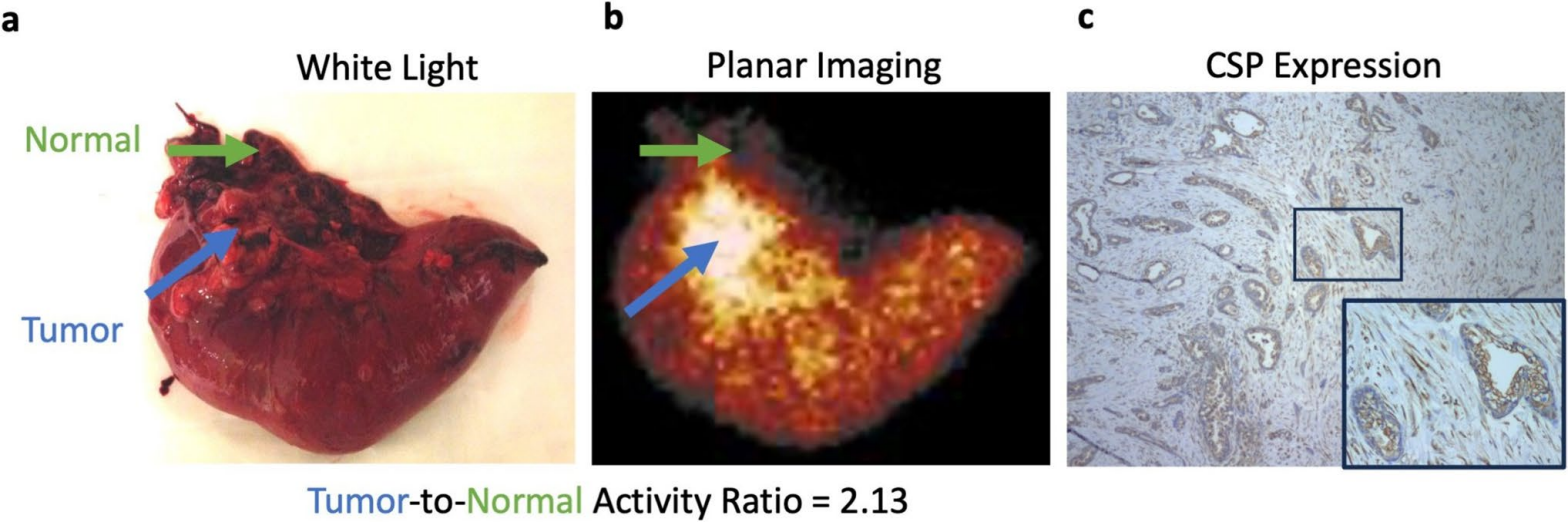
*Ex vivo* imaging and histological validation of PTP-01 uptake in Patient Pt 003. **a** *Ex vivo* planar scintigraphy of the resected specimen from Patient Pt 003, showing selective accumulation of PTP-01 in the tumor region. The tumor-to-background ratio was calculated based on the radiotracer uptake, confirming specific localization. **b** White light image of the resected specimen, with the tumor clearly demarcated. This image provides anatomical correlation with the *ex vivo* scintigraphy, confirming accurate tumor localization. **c** Histological analysis of the tumor tissue from Patient Pt 003, demonstrating CSP expression. The presence of CSP validates the specific binding of PTP-01 observed in both *in vivo* and *ex vivo* imaging. This confirms the feasibility of using PTP-01 for CSP-targeted imaging in pancreatic tumors

*Ex vivo* planar scintigraphy confirmed selective uptake, with a tumor-to-background ratio of 2.18 ± 0.14 (Fig. 3a, b). For Patient Pt 003, analysis included a white light image of the resected specimen and corresponding histological validation, confirming tumor localization and CSP expression (Fig. 3b, c). However, white light images and histological data were not systematically collected across all cases. Future studies should incorporate standardized resected specimen imaging and comprehensive histological validation. Histological validation of CSP expression was not systematically performed in this study, limiting direct confirmation of target presence in tumor tissues beyond the data available for Pt 003. However, prior preclinical studies have consistently demonstrated CSP expression in pancreatic tumors. Future investigations should include immunohistochemical analysis of resected tumors to correlate imaging findings with CSP expression at the cellular level. These results support PTP-01's specific targeting of CSP in pancreatic tumors.

### Pharmacokinetics Analysis

**Fig. 4:**
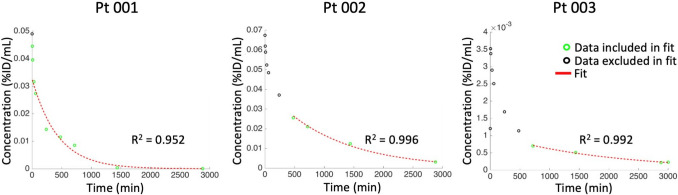
Plasma pharmacokinetics of PTP-01. Non-compartmental fit for each patient. Best fit simulation (red line) compared to quantitative image data (green circles for data included in fit and black circles for excluded data)

Blood and urine samples were collected at specified intervals post-PTP-01 administration to assess pharmacokinetics (Fig. 4). Sampling occurred at 1, 5, 10, 30, 60, 240, 480, 720, 1440, and 2880 min. Pharmacokinetic parameters were estimated using MATLAB, with curves generated via non-compartmental analysis, fitting the data tail to a single-exponential. The area under the curve (AUC) and area under the first moment curve (AUMC) were calculated using trapezoidal integration, with extrapolation from the last time point to infinity via closed-form numerical integration (Table 1).

**Table 1:** Pharmacokinetic parameters of PTP-01

Patient	T_1/2, β_ (h)	AUC (min *%ID/mL)	AUMC (min^2^ *%ID/mL)	MRT (min)	CL (mL/min)	CLR (mL/min)	V_ss_ (mL)
Pt 001	5.000	14.82	5918.62	399.38	6.75	1.47	2694.95
Pt 002	13.089	50.68	42042.80	829.52	1.97	N/A	1636.69
Pt 003	22.144	2.53	2639.58	1041.81	39.47	2.19	41119.10

Summary of pharmacokinetic parameters from non-compartmental analysis, including elimination half-life (T_1/2_); area under the curve (AUC); area under the first moment curve (AUMC); mean residence time (MRT); clearance (CL); renal clearance (CLR); volume of tissue distribution at steady state (V_ss_)

Notably, Patient Pt 003 displayed different pharmacokinetics compared to the other two patients, with blood concentrations approximately 50- to 70-fold higher and a time to maximum concentration (T_max_) of 5 min, compared to 1 min for the others.

The total urinary excretion of PTP-01 was 21.78% for Patient Pt 001 and 4.60% for Patient Pt 003. Unfortunately, urine samples were not collected for Patient Pt 002.

### CSP Density Estimation

**Fig. 5:**
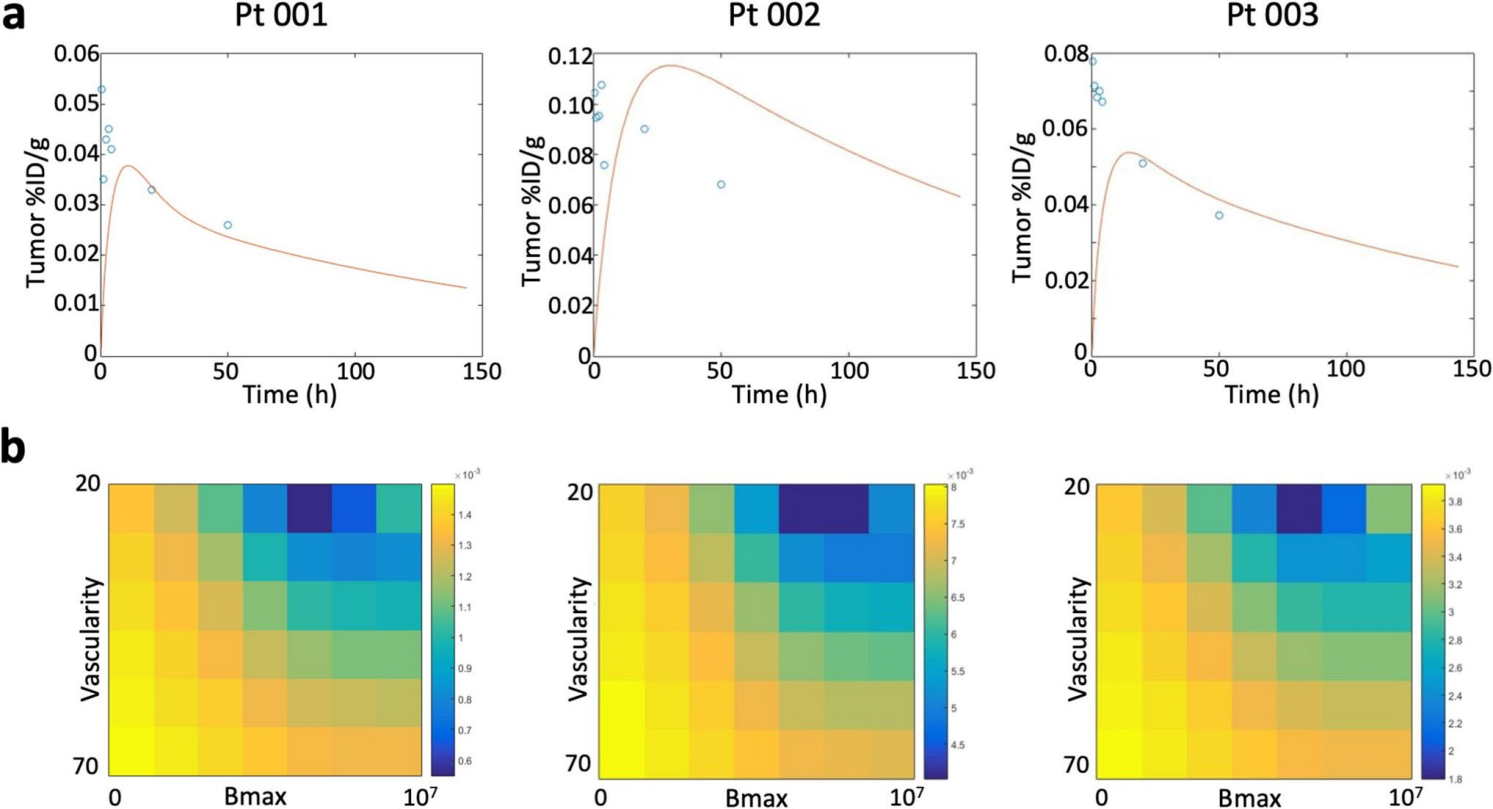
CSP Antigen Density Estimation. **a** *In vivo* CSP antigen density was estimated using the Krogh cylinder model. The best-fit simulation (red line) and quantitative imaging data (blue circles) are shown. **b** Mean squared error (MSE) map depicting the goodness-of-fit between the simulation and the quantitative imaging data, confirming the reliability of the model in estimating antigen density

CSP antigen density was estimated using the Krogh cylinder model, which provides insights into the spatial distribution of antigens within the tumor microenvironment (Fig. 5). All patients demonstrated between 0 and 10^7^ CSP molecules per cell, and capillary radii R varied between 20 and 70 µm. The model’s best-fit parameters suggested that Patients Pt 001 and Pt 003 exhibited an average CSP density of approximately 10^6^ CSP molecules per cell while Patient Pt 002 had 3 × 10^7^ CSP molecules per cell.

The Krogh cylinder model simulations were fitted to quantitative imaging data obtained from planar scintigraphy, with a good correlation observed between the predicted antigen densities and the experimental values. The mean squared error (MSE) between the simulation and the quantitative data confirmed the reliability of the model for estimating CSP density *in vivo* (Fig. 5b).

### PTP-01 Tolerability and Safety

The administration of PTP-01 was well-tolerated by all three patients, with no adverse events or significant safety concerns observed throughout the study. Patients experienced no immediate or delayed side effects, and safety monitoring, including clinical labs, ECGs, vital signs assessments, and physical examinations, revealed no abnormalities. This suggests that PTP-01 is a safe agent for further investigation.

## Discussion

### Relevance of CSP as a Target for Pancreatic Cancer

This first-in-human study establishes for the first time that CSP is a viable and bioavailable target in pancreatic cancer. CSP, typically localized intracellularly in healthy cells, is aberrantly expressed on the surface of cancer cells, providing an opportunity to specifically target tumor tissue while sparing normal cells. The ability of PTP-01 to selectively bind to CSP in pancreatic tumors highlights the potential of CSP as a cancer-specific biomarker.

The specific tumor uptake of PTP-01 observed in all three patients, evidenced by a tumor-to-background ratio greater than 2, further supports CSP’s role as a selective target. The dense stroma of pancreatic cancer has been purported to represent a barrier to the delivery of imaging and therapeutic agents. Interestingly, PTP-01 was able to penetrate into the tumor providing motivation for CSP as a target for radiopharmaceutical development and warranting further interrogation. More broadly, this study underscores the capability to exploit alterations in protein localization in cancer for imaging and ultimately for therapy. However, given the preliminary nature of the data, these findings should be interpreted cautiously, as larger studies are required to confirm the consistency and clinical significance of CSP targeting.

### PTP-01 Imaging Performance and Tumor-Specific Uptake

PTP-01 demonstrated specific uptake in pancreatic tumors across all patients, with a tumor-to-background ratio exceeding 2, as confirmed by *ex vivo* planar scintigraphy. This supports CSP-targeted imaging, though high background signals—particularly from the kidneys—limited tumor visibility on planar scintigraphy.

Although SPECT/CT imaging was conducted, these data are no longer accessible due to storage limitations over the 10-year period since the study. Only planar scintigraphy images used in original figures remain available. We acknowledge this as a significant limitation, as it precludes tomographic visualization of tumor-specific uptake. Quantitative analysis was performed at the time of acquisition using fused SPECT/CT images, but only planar data remain.

SPECT was chosen over PET due to practical and translational considerations. ^111^In-labeled radiotracers have a well-established role in oncologic imaging, and SPECT’s clinical accessibility made it a viable option for a first-in-human study. Additionally, ^111^In was used in preclinical development, ensuring a seamless transition to clinical evaluation. However, PET imaging offers superior sensitivity and quantification, and future studies should explore PET-compatible isotopes for improved imaging contrast and quantitative accuracy.

Despite these findings, several limitations warrant attention. The high kidney uptake observed significantly limited tumor visualization, underscoring the need to optimize the pharmacokinetics of PTP-01 to improve tumor-to-background contrast [20, 21]. This could involve modifying the peptide and adjusting the dosing regimen to enhance the tumor-to-kidney ratio. Alternative labeling strategies, such as PET-compatible isotopes (e.g., ⁶⁸Ga or ^1^⁸F), could also be explored to improve sensitivity and quantification, given PET’s superior resolution compared to SPECT.

Another limitation was the absence of systematic histological validation of CSP expression in resected tumors. Although CSP expression was evaluated during the study period, histological data remain available for only one patient due to archival sample loss. While this provides some validation, the lack of comprehensive histological correlation limits our ability to confirm PTP-01’s specificity for CSP in all cases. Future studies should incorporate systematic immunohistochemistry to better correlate imaging findings with target expression.

### Pharmacokinetic Behavior of PTP-01

The pharmacokinetic profile of PTP-01 provided valuable insights into its behavior within the human body. Blood and urine samples collected at multiple time points allowed us to estimate critical pharmacokinetic parameters such as T₁/₂, AUC, and MRT. These parameters help illustrate how long PTP-01 remains in circulation, its clearance rate, and its potential for sustained tumor imaging.

Interpatient variability was evident, particularly in the case of Patient Pt 003, who exhibited blood concentrations approximately 50- to 70-fold higher compared to the other two patients, along with a delayed Tmax of 5 min, compared to 1 min for Pt 001 and Pt 002. This variability could be due to differences in renal function, metabolic rates, or tumor characteristics, such as variations in vascularization, tumor burden, or CSP expression levels. The observed differences in urinary excretion between patients—Patient Pt 001 excreted 21.78% of the injected dose, while Patient Pt 003 excreted only 4.60%—suggest altered renal clearance may have contributed to the prolonged systemic circulation of PTP-01 in Pt 003. Additionally, the AUC, a measure of total drug exposure over time, and MRT indicate that PTP-01 has a sufficient window for imaging purposes. However, optimizing these pharmacokinetic parameters through personalized dosing strategies or agent modifications could further enhance its diagnostic utility, particularly in patients with atypical clearance profiles.

### CSP Antigen Density and Krogh Cylinder Model

The use of the Krogh cylinder model in this study was essential for estimating the density of CSP in pancreatic tumors and understanding its distribution within the tumor microenvironment [17, 18]. This model simulates PTP-01 diffusion from capillaries into tumors and binding to CSP, accounting for capillary permeability, interstitial diffusion, and binding kinetics. It provided insights into CSP spatial distribution *in vivo*. CSP density is crucial for both diagnostic and therapeutic applications. Higher CSP expression enhances PTP-01 specificity and imaging contrast. The model estimated CSP densities of 10⁶ molecules per cell in Patients Pt 001 and Pt 003, while Pt 002 exhibited 3 × 10⁷ molecules per cell. This variability underscores the need for patient-specific CSP quantification to determine the suitability of CSP-targeted imaging and therapy.

Quantifying CSP density aids in personalized treatment strategies. Not all pancreatic tumors express CSP at levels sufficient for targeting, making stratification essential. Patients with lower CSP expression may require alternative therapeutic approaches, while higher-expressing tumors may better respond to CSP-targeted interventions.

The integration of quantitative imaging with the Krogh model validated its accuracy in clinical scenarios. This model is an invaluable tool for optimizing imaging agent design and refining CSP-targeted therapies. However, it assumes uniform capillary distribution, which may not fully reflect tumor vascular heterogeneity. Additionally, capillary permeability and interstitial diffusion vary among patients. Future studies should incorporate immunohistochemistry or mass spectrometry to improve CSP density estimates across larger cohorts.

### Clinical Relevance and Future Implications

This study has significant clinical implications for CSP-targeted imaging and therapies in pancreatic cancer. Given the aggressive nature of PDAC, early detection and precise targeting are crucial for improving outcomes. The specific tumor uptake of PTP-01 in all three patients, with a tumor-to-background ratio > 2, highlights its potential to enhance diagnostic precision.

Phase 0 clinical trials play a critical role in developing imaging agents, offering early insights into biodistribution, pharmacokinetics, and safety [22]. In this study, Phase 0 evaluation of PTP-01 confirmed its tumor accumulation with no significant adverse events. Such trials help accelerate drug development by informing early “go/no-go” decisions, reducing the risk and cost of later-phase studies [23]. Additionally, Phase 0 trials are essential for optimizing dosing strategies and refining imaging protocols, ensuring a smooth transition to larger, more expensive clinical trials while minimizing development barriers [24]. This study reinforces the importance of Phase 0 trials in validating CSP-targeted imaging agents like PTP-01 for clinical use.

## Conclusion

Here we show proof-of-concept for a bench to clinical application for a new class of targets, proteins that are mislocalized to the plasma membrane in cancer. Specifically, CSP is druggable and abundant at an estimated 10^6 –^ 10^7^ CSP molecules per cell for use in targeted delivery. PTP-01 is a well-tolerated imaging agent with specific tumor uptake and slow kidney clearance. Overall, this study is the first to establish CSP as bioavailable and abundant in pancreatic cancer.

## Data Availability

The data supporting the findings of this study were collected over ten years ago as part of a first-in-human clinical trial (ClinicalTrials.gov Identifier: NCT01962909). Due to institutional data retention policies and storage limitations, most of the original raw data—including SPECT/CT images and full histological archives—are no longer accessible. However, selected processed datasets, including planar scintigraphy images, pharmacokinetic analyses, and CSP staining from one patient, are still available. Researchers interested in accessing the remaining non-identifiable data or discussing the methodology may contact the corresponding author upon reasonable request. Patient privacy protections and data limitations will be observed in accordance with IRB and institutional guidelines.
